# Clinical features and treatment outcomes of pediatric Langerhans cell histiocytosis with macrophage activation syndrome-hemophagocytic lymphohistiocytosis

**DOI:** 10.1186/s13023-022-02276-y

**Published:** 2022-04-04

**Authors:** Dong Wang, Xi-Hua Chen, Ang Wei, Chun-Ju Zhou, Xue Zhang, Hong-Hao Ma, Hong-Yun Lian, Li Zhang, Qing Zhang, Xiao-Tong Huang, Chan-Juan Wang, Ying Yang, Wei Liu, Tian-You Wang, Zhi-Gang Li, Lei Cui, Rui Zhang

**Affiliations:** 1 Hematology Center, Beijing Key Laboratory of Pediatric Hematology Oncology; National Key Discipline of Pediatrics, Capital Medical University; Key Laboratory of Major Diseases in Children, Ministry of Education; Beijing Children’s Hospital, Capital Medical University; National Center for Children’s Health, Beijing, China; 2 Hematologic Diseases Laboratory, Hematology Center, Beijing Pediatric Research Institute, Beijing Children’s Hospital, Capital Medical University, National Center for Children’s Health, Beijing, China; 3 Department of Pathology, Beijing Children’s Hospital, Capital Medical University, National Center for Children’s Health, Beijing, China; 4 Department of Hematology Oncology, Children’s Hospital Affiliated To Zhengzhou University, Zhengzhou University, Zhengzhou, China; 5 Beijing Children’s Hospital, Nanlishi Road No. 56, Xicheng District, Beijing, 100045 China

**Keywords:** Langerhans cell histiocytosis, Macrophage activation syndrome-hemophagocytic lymphohistiocytosis, BRAF-V600E mutation, Dabrafenib, Outcome

## Abstract

**Background:**

Langerhans cell histiocytosis (LCH) is a rare myeloid neoplasm. A few LCH patients had Macrophage activation syndrome-hemophagocytic lymphohistiocytosis (MAS-HLH), a life-threatening, hyper-inflammatory syndrome. We retrospectively described the clinical-biological characteristics of a series of 28 pediatric LCH patients with MAS-HLH in a single center. We further analyzed the difference in treatment outcomes between second-line chemotherapy (cytarabine and cladribine) and targeted therapy (dabrafenib) for BRAF-V600E-positive patients.

**Results:**

LCH patients with MAS-HLH were aged < 2 years, harbored high frequencies of risk organ, skin, or lymph nodes involvement, and most of them carried BRAF-V600E mutation in lesions (88.0%) or plasma (90.5%). Patients were firstly treated with the initial induction first-line therapy (vindesine-steroid combination), and most of them (26/28) failed to control the active MAS-HLH after one six-week course of induction treatment. Then they were shifted to second-line chemotherapy or targeted therapy dabrafenib. BRAF-V600E-mutant patients treated with dabrafenib had prompt resolution of MAS-HLH signs and symptoms with less toxicity than second-line chemotherapy. Moreover, the progression-free survival (PFS) rate for patients given dabrafenib was much higher than those treated with chemotherapy (4 year-PFS: 75% vs. 14.6%, *P* = 0.034).

**Conclusions:**

LCH patients with MAS-HLH harbored specific clinical-biology characteristics compared to the multisystem LCH without MAS-HLH. The BRAF inhibitor dabrafenib provides a promising treatment option for LCH with MAS-HLH.

**Supplementary Information:**

The online version contains supplementary material available at 10.1186/s13023-022-02276-y.

## Background

Langerhans cell histiocytosis (LCH) is a rare disease characterized by the accumulation of CD1a-positive (CD1a^+^)/CD207^+^ histiocytes with inflammatory lesions in various organ systems. The incidence of LCH ranges from 2.6 to 8.9 cases per million children, which was more prevalent than that in adults (estimated 1–2 cases per million adults) [1]. The clinical manifestations and prognosis of LCH are extremely variable, ranging from a solitary spontaneously regressing lesion to explosive multisystem disease with life-threatening organ dysfunction or permanent sequelae [2]. Since identifying recurrent mutations in the MAPK pathway, LCH has been considered a myeloid neoplastic disorder [3–6]. *BRAF*-V600E mutations have been identified in approximately 50% of LCH patients and were correlated with high-risk features of LCH and increased resistance to the first-line therapy [7, 8]. BRAF inhibitors have been demonstrated dramatic effects in the treatment of refractory or relapsed LCH patients who harbored *BRAF*-V600E mutations [9–12].

Hemophagocytic lymphohistiocytosis (HLH) is an immune-regulatory disorder characterized by excessive production of inflammatory cytokines. Patients present with multiple characteristic features including fever, cytopenia, hepatosplenomegaly, the elevation of typical HLH biomarkers, and can develop life-threatening multisystem organ dysfunction [13]. If left untreated, HLH has a high mortality rate. HLH can be categorized into two distinct forms: primary/familial HLH and secondary HLH. Familial HLH usually occurs in the presence of germline mutations in *PRF1*, *UNC13D*, *STX11*, *STXBP2*, and genes regulating lymphocyte cytotoxic activity [14]. Secondary HLH can be result from infections, malignancies, or autoimmune disease in the absence of any frank genetic predisposition to HLH [15]. Macrophage activation syndrome (MAS) is clinically characterized by pancytopenia, coagulopathy, hepatopathy, neurological disorders, and hemophagocytosis [16]. Secondary HLH associated with defined rheumatologic conditions is called MAS-HLH [17]. Several studies found that a few LCH patients had a presentation of MAS-HLH, and most of them had poor prognosis and a high risk of death [18–22]. LCH with MAS-HLH has been described in a few cases reports due to the rarity and high heterogeneity of LCH. Furthermore, the treatment response and outcomes for chemotherapy or targeted therapy have not been fully clarified. In this study, we described the clinical-biological characteristics of a series of pediatric LCH patients with MAS-HLH and analyzed the difference in treatment outcomes between targeted therapy (dabrafenib) and chemotherapy for BRAF-V600E-positive patients.

## Materials and methods

### Patients

Twenty-eight LCH patients (age < 18 years) with MAS-HLH referred to Beijing Children’s Hospital Hematology Center from Jan. 2016 to Dec. 2019 were enrolled in this study. The diagnosis of LCH was confirmed with the histological characteristic appearance of the LCH lesions on hematoxylin and eosin (HE)-stained sections and positive CD1a and/or Langerin staining of the lesional cells, taken from the most easily accessible but representative lesion [23]. Moreover, Patients must fulfill ≥ five of the eight diagnostic criteria for HLH according to the HLH-2004 protocol at diagnosis of LCH: fever, splenomegaly, cytopenia in ≥ 2 cell lineages, hypertriglyceridemia or hypofibrinogenemia, hyperferritinemia, elevated soluble CD25, hemophagocytosis in bone marrow or other tissue, low or absent NK-cell cytotoxicity [24].

### Therapeutic regimen

LCH patients were treated with a systemic chemotherapy regimen BCH-LCH 2014 (http://www.chictr.org.cn, identifier: ChiCTR2000030457), which was based on the LCH‑III and LCH s2005 protocol [25, 26]. The first-line therapy was a vindesine-steroid combination therapy. Patients were firstly treated with one or two six-week courses of initial induction therapy (vindesine 3 mg/m^2^/day IV bolus, once a week, for 6 weeks; prednisone 40 mg/m^2^/day orally, daily for 4 weeks afterward weekly reduction for 2 weeks), followed by the maintenance therapy (vindesine 3 mg/m^2^/day IV bolus, every 3 weeks; prednisone 40 mg/m^2^/day orally, day1-5, every 3 weeks; 6-mercaptopurine: 50 mg/m^2^/day orally, daily). The overall duration of the first-line therapy was 12 months.

From January 2016 to December 2017, patients with poorly controlled MAS-HLH were shifted to the second-line chemotherapy, which comprised four courses of intensive treatment arm A, four courses of arm B, and maintenance treatment. One 5-day course of arm A consisted of cytarabine (150 mg/m^2^/day IV guttae within 2 h, day 1–5), cladribine (9 mg/m^2^/day IV guttae, day 2–4), vindesine (3 mg/m^2^/day i.v. bolus, day 1) and dexamethasone (6 mg/m^2^/day, IV or orally, day 1–5).

The treatment arm B consisted of cytarabine, vindesine, and dexamethasone. One therapeutic course of arm A was administered every four weeks, while one course of arm B was performed every three weeks. The maintenance therapy included vindesine (3 mg/m^2^/day IV bolus, every 3 weeks), prednisone (40 mg/m^2^/day orally, day1–5, every 3 weeks), and 6-mercaptopurine (50 mg/m^2^/day orally, daily). The total duration of the second-line chemotherapy was 12 months.

From January 2018 to December 2019, BRAF inhibitor dabrafenib was administered to those patients who harbored BRAF-V600E mutation and had persistent active HLH after the initial induction therapy. Dabrafenib was given orally (2 mg/kg twice a day). The entire duration of dabrafenib was 12 months, adjusted according to disease assessment. Then patients were treated with maintenance chemotherapy including mercaptopurine, vindesine, and prednisone for six months [27].

### Clinical classification and evaluation of treatment response

Patients were stratified into three classifications according to the number of organs or systems involved and risk organs (RO: liver, spleen, and hematologic system) involvement: In single-system (SS) LCH, only one organ or system was involved without RO involvement. MS-LCH was defined as the involvement of two or more organs/systems with or without RO [25].

Treatment response was evaluated according to the International LCH Study Group Criteria [23]. Non-active disease (NAD), active disease (AD)/better, and AD/intermediate were defined as complete resolution, continuous regression of disease, or unchanged disease respectively. AD/worse was disease progression or appearance of some new lesions. Patients who responded to therapy were those who had NAD or AD/better response. Meanwhile, the quantitative Disease Activity Score (DAS) was retrospectively used as an evolution criterion [28]. Relapse was defined as the reappearance of signs and symptoms of active disease after either complete disease resolution or after a period of disease control that persisted for > 3 months on maintenance therapy [7]. Recovery of MAS-HLH indexes in this study was defined as that temperature < 37.4℃ for at least three days, hemoglobin ≥ 100 g/L, and platelets ≥ 100 × 10^9^/L, respectively.

### Detection of BRAF-V600E mutation

The presence of BRAF-V600E mutation in tissue or cell-free (cf) DNA was determined using the Digital PCR method, as described before [29]. The limit of the detection assay was determined at 0.1%.

### Statistical analysis

Differences between groups were tested with the Kruskal–Wallis or Mann–Whitney U test for quantitative variables and with Fisher’s exact test for qualitative variables. Progression-free survival (PFS) was estimated from the date of diagnosis or initial treatment until the date of one of the following events: progression, relapse, or death, whichever came first. The patients without event were censored at the date of the last contact. Survival rates were analyzed by the Kaplan–Meier method, and subgroups were compared with the log-rank test. All tests were performed using IBM SPSS 25.0 software (IBM Corp., USA). The cutoff date for analysis was September 30, 2021.

## Results

### Clinical features of LCH with MAS-HLH

A total of 518 consecutive patients with newly diagnosed LCH were enrolled in our center from Jan. 2016 to Dec. 2019. Two hundred and eighty-one (54.2%) patients were SS-LCH and 237 (45.8%) patients were MS-LCH. Twenty-eight (5.4%) of them had MAS-HLH at the time of LCH diagnosis, all of whom presented with MS-LCH. There were 15 (53.6%) boys and 13 (46.4%) girls in the patients with MAS-HLH. Comparing of clinical features between MS-LCH patients with or without MAS-HLH (Table 1), patients with MAS-HLH were typically younger than those without MAS-HLH (*P* < 0.001), and all the patients with MAS-HLH were aged < 2 years. Moreover, the patients with MAS-HLH harbored higher frequencies of liver, spleen, skin, or lymph nodes involvement (*P* values were < 0.001, < 0.001, < 0.001, and 0.005, respectively). More patients with MAS-HLH carried BRAF-V600E mutation in lesions or plasma with compared to the patients without MAS-HLH (lesions: 88.0% vs. 63.7%, *P* = 0.019; plasma: 90.5% vs. 49.0%, *P* < 0.001, respectively).

**Table 1:** Comparison of clinical-biological characteristics in MS-LCH patients with (n = 28) or without (n = 209) MAS-HLH in this study

Clinical characteristics	MS-LCH	Without MAS-HLH	With MAS-HLH	*P* values
n	237	209	28	
Sex				
Male	141 (59.5%)	126 (60.3%)	15 (53.6%)	0.542
Female	96 (40.5%)	83 (39.7%)	13 (46.4%)	
Age at diagnosis (years)				
≥ 2	105 (44.3%)	105 (50.2%)	0	< 0.001
< 2	132 (55.7%)	104 (49.8%)	28 (100%)	
Median (range)	1.70 (0.10–14.95)	2.0 (0.10–14.95)	1.0 (0.20–1.78)	< 0.001
Bone involvement				
No	40 (16.9%)	34 (16.3%)	6 (21.4%)	0.590
Yes	197 (83.1%)	175 (83.7%)	22 (78.6%)	
Skin involvement				
No	106 (44.7%)	102 (48.8%)	4 (14.3%)	< 0.001
Yes	131 (55.3%)	107 (51.2%)	24 (85.7%)	
Liver involvement				
No	152 (64.1%)	147 (70.3%)	5 (17.9%)	< 0.001
Yes	85 (35.9%)	62 (29.7%)	23 (82.1%)	
Spleen involvement				
No	183 (77.2%)	180 (86.1%)	3 (10.7%)	< 0.001
Yes	54 (22.8%)	29 (13.9%)	25 (89.3%)	
Hematologic involvement				
No	199 (84.0%)	199 (95.2%)	0	< 0.001
Yes	38 (16.0%)	10 (4.8%)	28 (100%)	
Lung involvement				
No	158 (66.7%)	141 (67.5%)	17 (60.7%)	0.524
Yes	79 (33.3%)	68 (32.5%)	11 (39.3%)	
Pituitary involvement				
No	186 (78.5%)	163 (78.0%)	23 (82.1%)	0.807
Yes	51 (21.5%)	46 (22.0%)	5 (17.9%)	
Lymph nodes involvement				
No	188 (79.3%)	172 (82.3%)	16 (57.1%)	0.005
Yes	49 (20.7%)	37 (17.7%)	12 (42.9%)	
BRAF-V600E in lesion tissues^a^				
Total available samples	160	135	25	
Negative	52 (32.5%)	49 (36.3%)	3 (12.0%)	0.019
Positive	108 (67.5%)	86 (63.7%)	22 (88.0%)	
BRAF-V600E in plasma cell-free DNA^a^				
Total available samples	164	143	21	
Negative	75 (45.7%)	73 (51.0%)	2 (9.5%)	< 0.001
Positive	89 (54.3%)	70 (49.0%)	19 (90.5%)	

MS-LCH, multisystem Langerhans cell histiocytosis; MAS, macrophage activation syndrome; HLH, hemophagocytic lymphohistiocytosis

^a^BRAF-V600E mutation in biopsies of lesion tissue or plasma cell-free DNA was determined in 160 and 164 MS-LCH patients respectively

### Biologic and laboratory parameters of patients

All the 28 patients with MAS-HLH had the histological examinations for a definitive diagnosis of LCH, with the biopsy specimens taken from the skin (24 cases), bone (3), or liver (1). The typical LCH cells are large, round to oval shape, with pale cytoplasm and reniform or coffee-bean nuclei on HE staining (Additional file 1: Figure S1A). The crucial element for diagnosing LCH is biopsy with the characteristic histiocytes with positive CD1a and/or CD207 (langerin) staining (Additional file 1: Figure S1B-C). Furthermore, only one patient had the characteristic appearance of LCH lesions in biopsy specimens of bone marrow, of which CD1a and CD207 staining were positive (Additional file 1: Figure S1D-E). A few surface markers, including CD163, fascin, and factor XIII, are helpful to distinguish mixed histiocytic lesions (e.g., Erdheim-Chester or juvenile xanthogranuloma) [30].

**Fig. 1:**
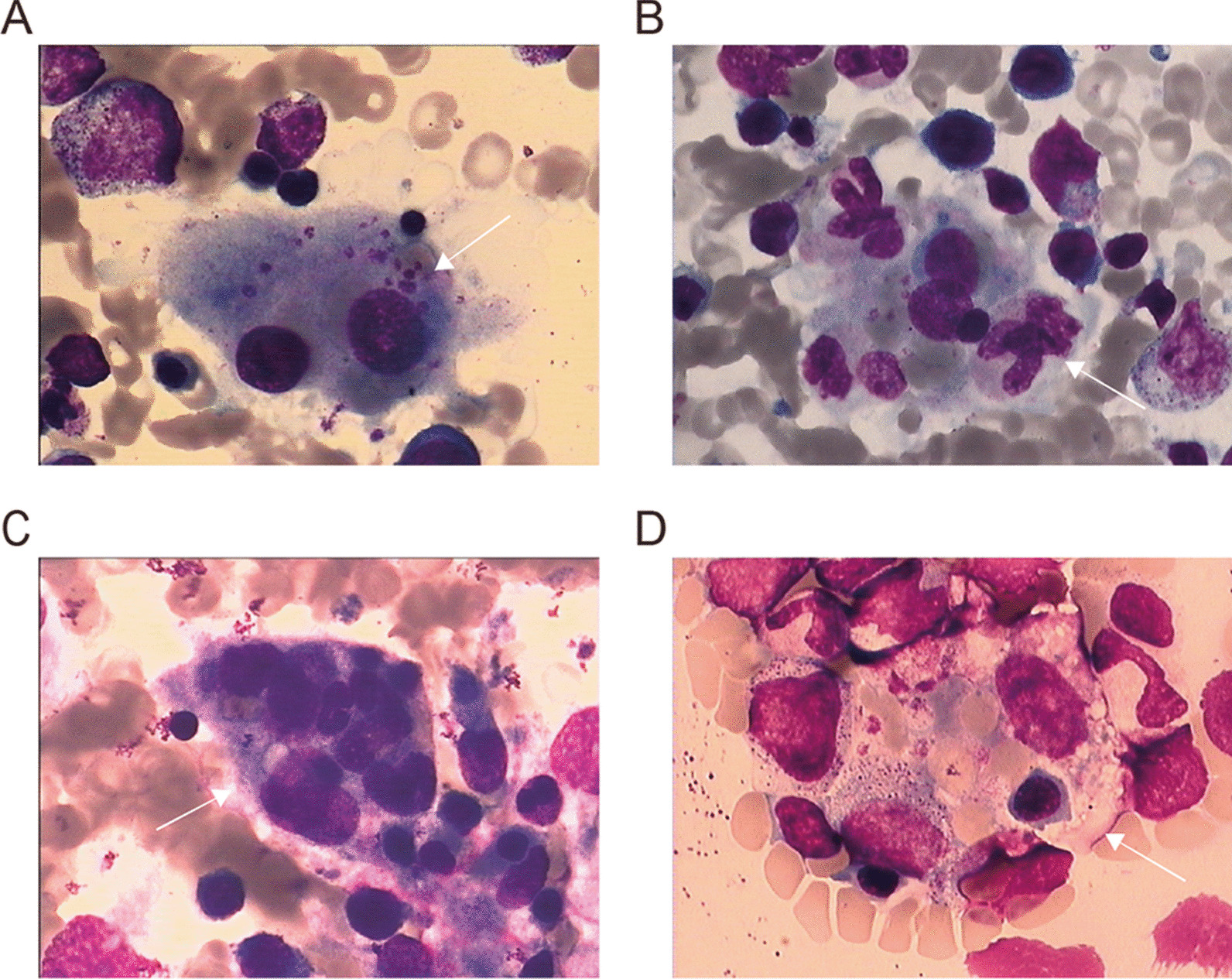
Presence of hemophagocytosis in bone marrow (arrowheads)

All the patients with MAS-HLH met at least 5 of the 8 HLH diagnostic criteria, as shown in Table 2 and Additional file 1: Table S1. All of them had a fever and greatly increased sCD25 level. Most patients (89.3%) had cytopenia in at least two lineages (mainly affecting erythroid or megakaryocytes) and splenomegaly. 72.0% of the evaluable patients had low or absent NK cell activity. Approximately half of the patients had hypertriglyceridemia and hypofibrinogenemia. However, only 14.3% of the patients have high ferritin (> 500 ng/mL). 46.4% had the presence of hemophagocytosis in bone marrow (Fig. 1).

**Table 2:** HLH-associated laboratory parameters in LCH patients with MAS-HLH

Laboratory parameters	Normal range	MS-LCH with MAS-HLH, Median (Range) or n (%)
Whole blood counts		
Hemoglobin (g/L)	110–160	70.0 (49.0–99.0)
90–70	–	15 (53.6%)
< 70	–	13 (46.4%)
Platelets (× 10^9^/L)	100–400	49.5 (1.0–422.0)
≥ 100	–	3 (10.7%)
< 100	–	25 (89.3%)
Neutrocytes (× 10^9^/L)	0.7–4.6	2.5 (0.7–17.7)
≥ 1.0	–	19 (67.9%)
< 1.0	–	9 (32.1%)
Cytopenia (≥ 2 cell lineages)		
Yes	–	25 (89.3%)
No	–	3 (10.7%)
HLH indexes, N (%)		
Hypertriglyceridemia > 3 mmol/L	0.4–1.7	15 (53.6%)
Hypofibrinogenemia < 1.5 g/L	2.0–4.0	17 (60.7%)
Hyperferritin > 500 ng/mL	6–159	4 (14.3%)
Soluble CD25 > 6400 pg/mL	< 6400	28 (100%)
Low or absent NK cell activity^a^	≥ 15.11%	18 (72.0%)
Hemophagocytosis in bone marrow	–	13 (46.4%)
Fever (T > 38.5℃, ≥ 7 days)	< 37.4	28 (100%)
Splenomegaly	–	26 (92.9%)
Inflammatory and cytokine indexes		
ESR (mm/h)	0–15	2.5 (1.0–104.0)
CRP (mg/L)	0–10	45.0 (5.0–139.0)
γ-IFN (pg/mL)	1.6–17.3	1.8 (0–44.7)
TNF (pg/mL)	0.1–5.2	1.29 (0–70.5)
IL-10 (pg/mL)	2.6–4.9	11.6 (3.3–77.6)
IL-6 (pg/mL)	1.7–16.6	59.3 (2.4–1356.1)
Liver function indexes		
TBIL (µmol/L)	3.4–20.5	17.3 (4.4–45.3)
ALB (g/L)	35–55	29.0 (16.1–37.7)
ALT (U/L)	5–40	12.8 (3.5–143.7)
AST (U/L)	5–40	32.6 (10.1–203.2)
r-GT (U/L)	5–50	28.8 (5.0–293.7)
LDH (U/L)	110–295	317.5 (106.0–988.0)

LCH, multisystem Langerhans cell histiocytosis; HLH, hemophagocytic lymphohistiocytosis; MAS, macrophage activation syndrome; NK cell, natural killer cell; ESR, erythrocyte sedimentation rate; CRP, C-reactive protein; γ-IFN, γ-Interferon; TNF, tumor necrosis factor; IL, Interleukin; TBIL, total bilirubin; ALB, serum albumin; ALT, alanine aminotrans; AST, aspartate transaminase; r-GT, gamma glutamyltranspeptidase; LDH, lactate dehydrogenase

^a^25 patients with available data regarding NK cell activity

As to the inflammatory biologic parameters, the median levels of CRP, IL-10, and IL-6 were elevated in LCH with MAS-HLH, compared to the reference values. The median levels of liver function indexes were normal, except for the increased LDH. In addition, all patients were tested for infection, including Epstein-Barr virus (EBV), cytomegalovirus, human herpes simplex virus, the rubella virus, and toxoplasma at the time of diagnosis, and none of the patients had these infections.

### Treatment response of MAS-HLH signs and symptoms

Twenty-two (88.0%) of the 25 patients with MAS-HLH were BRAF-V600E mutation-positive, three were mutation-negative, and another three were not assessable for BRAF status. All of the 28 patients were firstly treated with the initial induction treatment based on the vindesine-prednisone combination. Only two patients (7.1%) with positive BRAF-V600E mutation had the improvement of MAS-HLH and LCH lesions after one six-week course of induction therapy, and they were kept to be treated with the first-line chemotherapy. Twenty BRAF-V600E-mutant patients with the presence of active MAS-HLH were shifted to the salvage therapy or targeted therapy: eight patients received the second-line chemotherapy including cladribine and cytarabine, and twelve patients were given with dabrafenib. Detailed information about the study cohorts of patients is summarized in Figure S2.

**Fig. 2:**
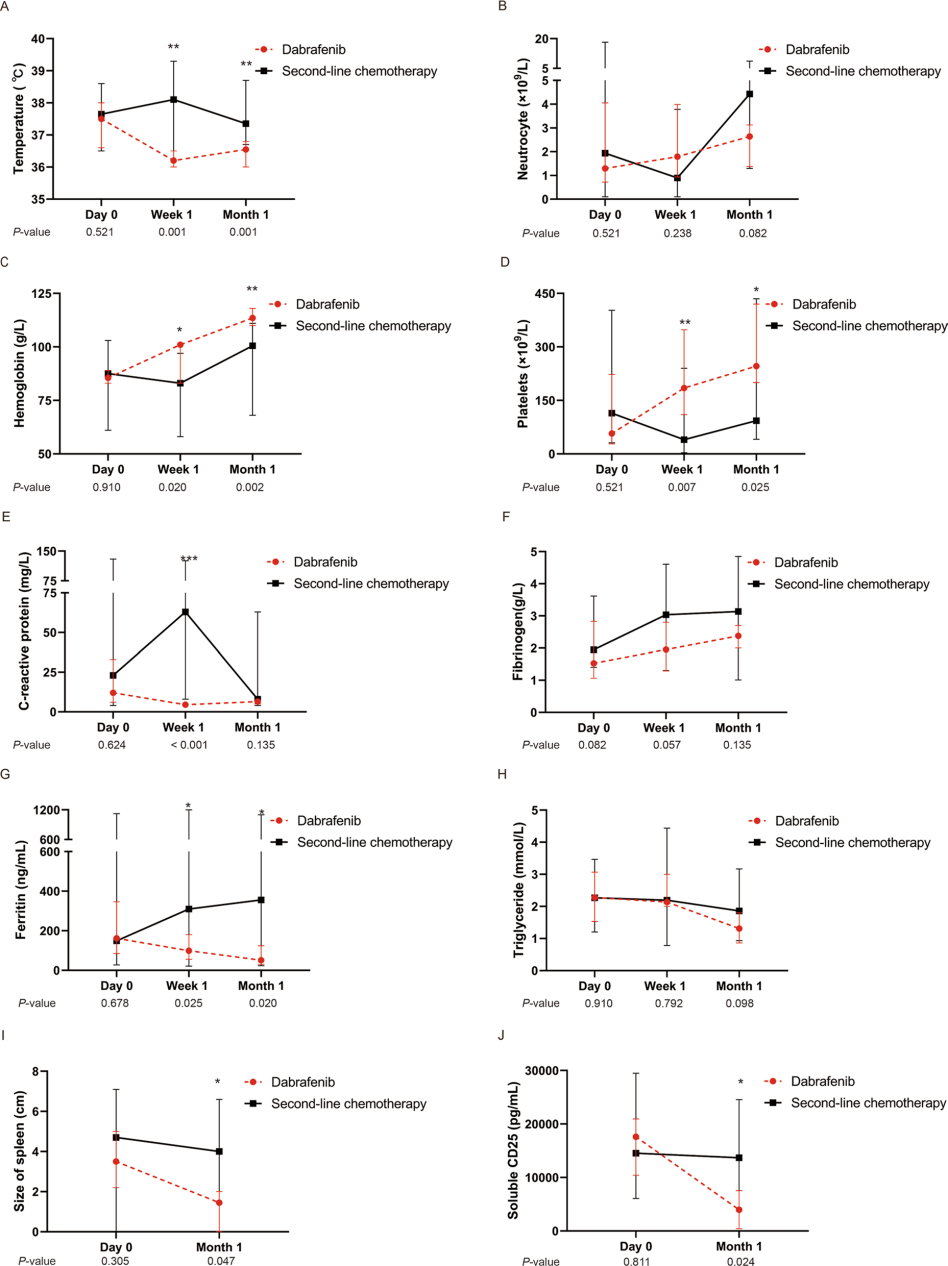
Comparison of treatment response of MAS-HLH indexes between patients treated with dabrafenib and second-line chemotherapy. *P < 0.05, **P < 0.01, ***P < 0.001

**Fig. 3:**
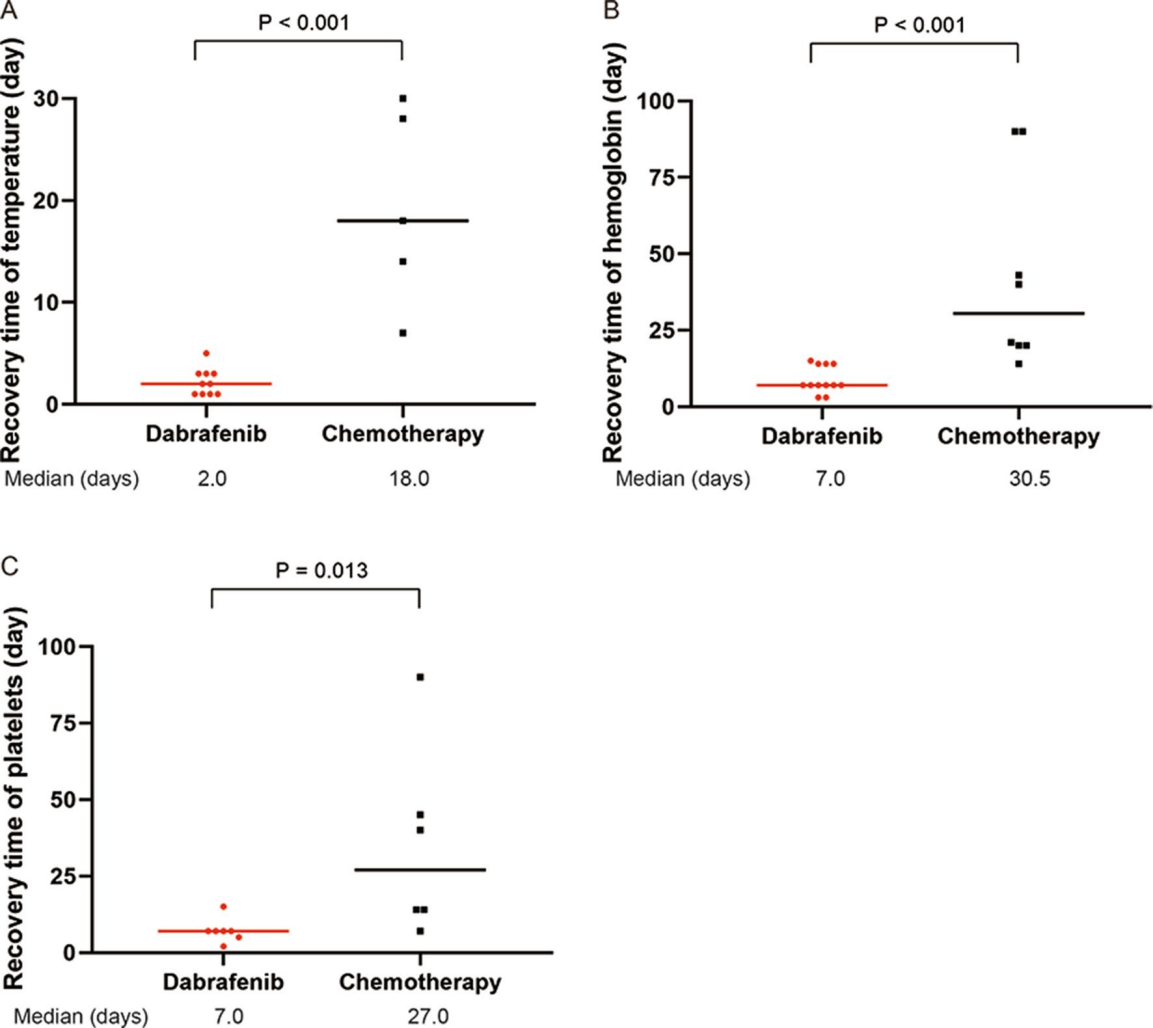
Comparison of the recovery time between patients treated with dabrafenib and second-line chemotherapy. **A** Body temperature; **B** hemoglobin; **C** platelets

Compared to the eight patients treated with the second-line chemotherapy, the 12 patients experienced more rapid resolution of MAS-HLH after dabrafenib initiation (Fig. 2). The MAS-HLH indexes including fever, cytopenia, CRP level, and hyperferritin were significantly improved in one week (*P* values were 0.001, 0.020, < 0.001, 0.025, respectively); the size of the spleen and the level of sCD25 were significantly decreased in one month (*P* values were 0.047 and 0.024, respectively) after dabrafenib administration. The recovery time of temperature, hemoglobin, and platelets of patients was significantly shorter in the dabrafenib group than in the chemotherapy group (Fig. 3, *P* values were < 0.001, < 0.001, and 0.013, respectively), indicating the more rapid treatment responses in patients with dabrafenib. According to the evaluation criteria of the Histiocyte Society, all the 12 patients were evaluated as AD-better response after one month of dabrafenib administration, while 3 of the 8 (37.5%) patients had AD-better response of MAS-HLH signs and LCH lesions after the first two courses (5 weeks) of chemotherapy (*P* = 0.004). Furthermore, we compared the DAS after dabrafenib treatment and chemotherapy (Additional file 1: Figure S3). The median DAS decreased from 12.5 at the initiation of dabrafenib to 2.5 on month 1 (*P* < 0.001), and the DAS also had a slight decline from 12.0 at the beginning of chemotherapy to 8.5 after two therapeutic courses (*P* = 0.038). Of note, the decline was more remarkable for dabrafenib than for chemotherapy, indicating a better treatment response for dabrafenib.

In addition, three patients without BRAF-V600E and two patients with unavailable BRAF status were treated with the second-line chemotherapy after the failure of induction treatment. After the four courses of intensive treatment, one patient with the negative mutation and two patients with unknown BRAF had AD-better response of both MAS-HLH and LCH lesions. One patient with negative- BRAF-V600E got the resolution of MAS-HLH, but had progression of the pituitary at that time. One patient without the mutation experienced HLH progression and died of intracranial hemorrhage after the two courses of intensive treatment.

### Relapse and survivals

**Fig. 4:**
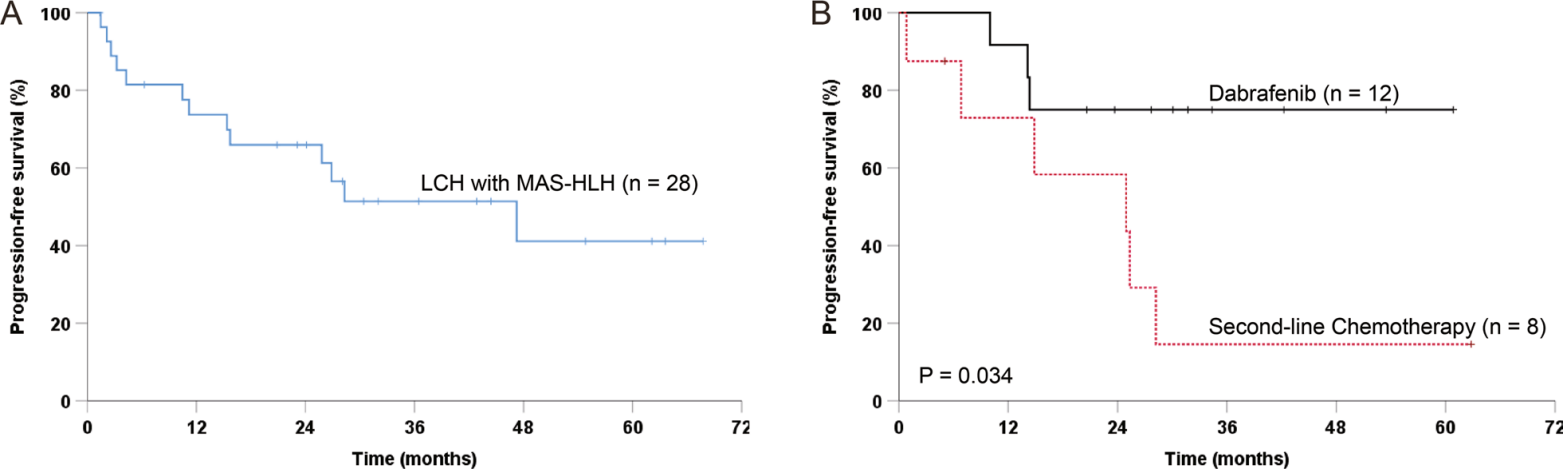
Progression-free survival rates for LCH patients with MAS-HLH. **A** For the whole cohort. **B** Comparison for patients treated with dabrafenib or second-line chemotherapy

Since diagnosis, the median follow-up duration for all the 28 patients was 25.0 months (range, 1.4 to 67.8 months). Twenty-one patients have completed all treatments, with the median observation time of 12.4 months (range, 1.4 to 53.4 months) since the end of therapy. The patients were alive until the last follow-up, except one, who died of MAS-HLH progression 2.8 months after admission. However, 12 (42.8%) patients experienced progression or relapse with a median time of 13.3 months later, and the PFS at four years was 41.1% ± 12.4% (Fig. 4A). The most frequent site of progression/relapse was bone (7 cases), and the other sites included liver (2), pituitary (1), spleen (1), and hematologic system (1).

The median duration of follow-up since the beginning of secondary therapy was 28.9 (range, 10.0–60.8) for dabrafenib and 19.9 (range, 0.8–62.8) for the second-line chemotherapy, respectively (*P* = 0.238). During the follow-up, three of 12 (25.0%) patients given dabrafenib progressed or relapsed, while six of eight (75.0%) patients treated with the second-line chemotherapy experienced progression or relapse (*P* = 0.065). Moreover, there was a significantly increased PFS in patients who received dabrafenib compared to those treated with chemotherapy. (4 year-PFS: 75.0% ± 12.5% vs. 14.6% ± 13.5%, *P* = 0.034; Fig. 4B).

Notably, the load of cfBRAF-V600E was monitored in nine patients during dabrafenib administration. The median level of cfDNA decreased from 10.0% to 0.45% following one month of dabrafenib, but 87.5% of them remained positive for 12 months (Additional file 1: Table S2).

### Adverse events

The principal acute toxicity for patients treated with second-line chemotherapy was myelosuppression and 12 of the 13 (92.3%) patients experienced profound pancytopenia (Common Terminology Criteria for Adverse Events CTCAE grade 3–4), which complicated by severe infection and fever. By contrast, twelve adverse events (AEs) occurred in 4 of 12 patients (33.3%) during dabrafenib administration. The AEs were predominantly skin-related toxicity (9/12, 75.0%), and most of them were skin rashes. The nondermatologic AEs included diarrhea, vomiting, fatigue, joint pain, and transient myocardium enzyme rising. All the AEs were at grade 1 or 2.

## Discussion

This study retrospectively described the clinical features and treatment outcomes of pediatric LCH with MAS-HLH in a single center. Our data showed that LCH patients with MAS-HLH were very young (< 2 years), harbored higher frequencies of RO, skin, or lymph nodes involvement, and most of them carried BRAF-V600E mutation in lesions or plasma. When evaluated for treatment outcomes, BRAF-V600E positive-patients treated with BRAF-specific inhibitor dabrafenib had prompt resolution of MAS-HLH signs and symptoms than the second-line chemotherapy. Furthermore, the PFS for patients given dabrafenib was significantly higher than those treated with the chemotherapy.

MAS-HLH is a life-threatening presentation of LCH. It is proposed that LCH lesions are infiltrated with many dysfunctional T cells and other inflammatory cells, which exuberantly produce many pro-inflammatory cytokines and chemokines [8, 31]. These cytokines, such as interferon γ, tumor necrosis factor, and interleukin 2, could drive the activation of macrophages and T cells and induce hyper-inflammation [18]. MAS-HLH usually occurred in LCH patients with active lesions and multiple RO involved [19]. We also found that MAS-HLH was strongly associated with infants with high-risk RO-positive LCH. We noted that MAS-HLH was correlated with the involvement of skin or lymph nodes and the BRAF-V600E mutation, which were consistent with the severity of the disease.

In most previous studies, LCH patients with MAS-HLH had a poor prognosis. If treated by chemotherapy (LCH- or HLH-directed therapy) or received hematopoietic stem cell transplantation), the overall survival rate of patients with MAS-HLH was significantly lower than that of patients without MAS-HLH (68.9% vs 97.1%; *P* < 0.0001) [18]. Moreover, those patients meeting HLH criteria who received HLH-directed therapy demonstrated a trend toward poorer 5-year survival compared with those who received LCH-directed therapy. In this study, all LCH patients with MAS-HLH were treated with LCH-directed therapy, most (26/28) patients were alive, and the active HLH and LCH disease were controlled by the targeted therapy or chemotherapy. Although these patients were not detected for the familial HLH gene mutation, the MAS-HLH was considered a special presentation of LCH due to the efficacy of LCH-directed therapy.

Our data showed that salvage chemotherapy including cytarabine and cladribine was effective in controlling active MAS-HLH and LCH. Still, it was associated with high toxicity and required extensive supportive care. The majority of those patients had grades 3–4 of AEs, which were intolerable and life-threatening for the young patients with MAS-HLH. LCH patients with MAS-HLH were usually younger than 2 years of age, have RO involvement, and frequently harbored the BRAF-V600E mutation. Therefore, BRAF inhibitors were considered to apply in those pediatric patients. Lee et al. reported four LCH infants with MAS-HLH treated with dabrafenib and showed that all patients achieved completed clinical responses by eight weeks of therapy [22]. Similarly, our findings showed that dabrafenib had a noticeable rapid effect on MAS-HLH and could control LCH disease effectively with less toxicity than chemotherapy.

The targeted therapy provides a promising treatment option for refractory or relapsed LCH over recent years [9, 10, 27]. However, the targeted drugs seemed unable to eradicate tumor clones clearly, and relapse after discontinuation of targeted therapy remained a significant obstacle for improving the prognosis of patients. The cf BRAF-V600E remained positive for the most (9/12) patients after six months of vemurafenib administration, and persistent positive of cfDNA was closely associated with relapse [10]. We performed a longitudinal evaluation of cfBRAF-V600E during dabrafenib treatment; 87.5% of patients with MAS-HLH remained positive for 12 months. It is proposed that this type of high-risk LCH arises from somatic mutation of hematopoietic progenitors, and it is hard to eradicate the underlying precursor cells with targeted drugs alone. The relatively low relapse rate (25.0%) in this study might due to the short follow-up time after dabrafenib discontinuation.

## Conclusions

In conclusion, the present study described the clinical-biology features of pediatric LCH with MAS-HLH, including young age, RO involvement, and high positivity of BRAF-V600E mutation. Moreover, we showed that BRAF inhibitor dabrafenib could quickly and effectively control MAS-HLH and improve the LCH condition compared to the chemotherapy. The challenge exists in achieving sustained resolution of LCH disease, and further prospective studies with large sample sizes and extended follow-up are needed.

## Supplementary Information

**Additional file 1: Table S1.** Clinical characteristics and biologic parameters of LCH patients with MAS-HLH. **Table S2.** Longitudinal evaluation of cell-free BRAF-V600E during dabrafenib treatment. **Figure S1.** Histology of LCH lesions obtained from a skin biopsy (A-C) or a bone marrow biopsy (D-F) in the LCH patients with MAS-HLH. (A) and (D): HE staining; (B) and (E): CD1a-positive immunostaining; (C) and (F): CD207 (langerin)-positive immunostaining. **Figure S2.** Study cohorts of pediatric LCH and MAS-HLH. **Figure S3.** Comparison of DAS after one month of dabrafenib and five weeks (two therapeutic courses) of second-line chemotherapy.

## Data Availability

The datasets used and/or analyzed during the current study are available from the corresponding author on reasonable request.

## Abbreviations

AD: Active disease
EBV: Epstein-Barr virus
MAS: Macrophage activation syndrome
HLH: Hemophagocytic lymphohistiocytosis
LCH: Langerhans cell histiocytosis
MS: Multisystem
NAD: Non-active disease
PFS: Progression-free survival
RO: Risk organs
