# Supplementary material for: Clinical features and treatment outcomes of pediatric Langerhans cell histiocytosis with macrophage activation syndrome-hemophagocytic lymphohistiocytosis
Source: Orphanet J Rare Dis. 2022 Apr 4;17:151. doi: 10.1186/s13023-022-02276-y (PMC8981711; doi:10.1186/s13023-022-02276-y)
Supplement: Supplementary file 1 — Additional file 1: Table S1. Clinical characteristics and biologic parameters of LCH patients with MAS-HLH. Table S2. Longitudinal evaluation of cell-free BRAF-V600E during dabrafenib treatment. Figure S1. Histology of LCH lesions obtained from a skin biopsy (A-C) or a bone marrow biopsy (D-F) in the LCH patients with MAS-HLH. (A) and (D): HE staining; (B) and (E): CD1a-positive immunostaining; (C) and (F): CD207 (langerin)-positive immunostaining. Figure S2. Study cohorts of pediatric LCH and MAS-HLH. Figure S3. Comparison of DAS after one month of dabrafenib and five weeks (two therapeutic courses) of second-line chemotherapy. [file 13023_2022_2276_MOESM1_ESM.docx]

Table S1 Clinical characteristics and biologic parameters of LCH patients with MAS-HLH

| Case No. | Sex | Age (Years) | Organ involvements | BRAF-V600E in lesion tissues | cell-free BRAF-V600E in plasma | HLH indexes | | | | | | | | |
| --- | --- | --- | --- | --- | --- | --- | --- | --- | --- | --- | --- | --- | --- | --- |
|  |  |  |  |  |  | Fever | Splenomegaly | cytopenia | Triglyceride  (mmol/L) | Fibrinogen  (g/L) | Ferritin  (ng/ml) | sCD25  (pg/ml) | NK cells activity (%) | Hemophagocytosis |
| 1 | Female | 1.00 | Bones, hematopoietic system, pituitary | Positive | Positive | Yes | Yes | Yes | 1.87 | 3.58 | 84.50 | 16151 | 13.53 | No |
| 2 | Male | .70 | Bones, skin, liver, spleen, hematopoietic system,lung, lymph nodes, pituitary | Positive | Positive | Yes | No | Yes | 3.34 | .82 | 578.20 | 37632 | 15.53 | Yes |
| 3 | Male | .20 | Skin, spleen, hematopoietic system, lymph nodes | Positive | Positive | Yes | Yes | Yes | 3.21 | .59 | 311.90 | 35604 | NA | No |
| 4 | Male | 1.20 | Bones, skin, liver, spleen, hematopoietic system, lung | Positive | Positive | Yes | Yes | Yes | 3.22 | .82 | 193.10 | 27090 | 10.84 | Yes |
| 5 | Female | 1.10 | Bones, skin, spleen, hematopoietic system, lung, lymph nodes, pituitary | Positive | Positive | Yes | Yes | No | 1.44 | 4.86 | 114.00 | 24649 | 12.46 | No |
| 6 | Male | .60 | Bones, skin, liver, spleen, hematopoietic system, lung, thymus, lymph nodes | Positive | Positive | Yes | Yes | Yes | 3.23 | 1.00 | 45.30 | 21981 | 16.10 | Yes |
| 7 | Male | .50 | Bones, skin, liver, spleen, hematopoietic system, lung | Positive | Positive | Yes | Yes | Yes | 3.07 | 1.05 | 45.50 | 7510 | 14.83 | Yes |
| 8 | Female | 1.10 | Bones, skin, liver, spleen, hematopoietic system, lung | Positive | Positive | Yes | Yes | Yes | 2.15 | 1.90 | 118.90 | 11124 | 14.11 | No |
| 9 | Female | .84 | Bones, skin, liver, spleen, hematopoietic system, lung, lymph nodes | Positive | Positive | Yes | Yes | Yes | 4.58 | .77 | 79.50 | 22803 | 14.49 | Yes |
| 10 | Male | 1.01 | Skin, liver, hematopoietic system, lung, ear | Positive | Positive | Yes | Yes | Yes | 2.43 | 1.26 | 50.70 | 14242 | 11.46 | Yes |
| 11 | Male | .42 | Skin, liver, spleen, hematopoietic system, lymph nodes, ear | Positive | Positive | Yes | Yes | Yes | 4.25 | 1.61 | 274.60 | 12415 | 14.53 | No |
| 12 | Male | 1.00 | Skin, liver, hematopoietic system | Positive | NA | Yes | Yes | Yes | 3.16 | 1.65 | 31.80 | 10482 | 14.47 | No |
| 13 | Female | 1.43 | Bones, spleen, hematopoietic system, eye | Positive | Positive | Yes | Yes | Yes | 3.26 | 1.66 | 160.40 | 13530 | 17.11 | No |
| 14 | Female | 1.50 | Bones, skin, liver, spleen, hematopoietic system, lung, lymph nodes | Positive | Positive | Yes | Yes | Yes | 1.74 | .89 | 100.00 | 12966 | 15.08 | Yes |
| 15 | Female | .67 | Bones, skin, liver, spleen, hematopoietic system, lung, pituitary, ear | Positive | Positive | Yes | Yes | Yes | 4.04 | 1.58 | 485.30 | 10950 | 17.01 | No |
| 16 | Male | 1.65 | Bones, skin, liver, spleen, hematopoietic system, pituitary | Positive | NA | Yes | Yes | Yes | 2.63 | .62 | 327.00 | 26630 | 11.42 | Yes |
| 17 | Female | 1.24 | Bones, skin, liver, spleen, hematopoietic system, lung, ear | Positive | Positive | Yes | Yes | Yes | 2.98 | 1.00 | 974.00 | 11985 | NA | No |
| 18 | Male | 1.65 | Bones, skin, liver, spleen, hematopoietic system, lymph nodes | Positive | NA | Yes | Yes | Yes | 1.53 | 1.18 | 36.30 | 18993 | 16.08 | Yes |
| 19 | Male | 1.54 | Bones, skin, liver, spleen, hematopoietic system, ear, eye | Positive | Positive | Yes | Yes | Yes | 3.77 | 2.16 | 182.70 | 10260 | 17.06 | Yes |
| 20 | Female | 1.78 | Bones, skin, liver, spleen, hematopoietic system, lung | Positive | Positive | Yes | Yes | Yes | 2.04 | 1.30 | 4.60 | 15110 | 13.62 | Yes |
| 21 | Female | 1.51 | Bones, skin, spleen, hematopoietic system, lymph nodes, eye | Positive | Positive | Yes | Yes | Yes | 3.14 | 1.87 | 239.50 | 8807 | 12.26 | No |
| 22 | Female | .44 | Bones, skin, liver, spleen, hematopoietic system, lung, lymph nodes | Positive | Positive | Yes | Yes | Yes | 2.44 | .80 | 392.50 | 19056 | 12.75 | No |
| 23 | Male | .80 | Bones, liver, spleen, hematopoietic system, lung, lymph nodes | Negative | Negative | Yes | Yes | No | 4.37 | 1.22 | 88.40 | 37846 | 13.25 | No |
| 24 | Male | .40 | Bones, liver, hematopoietic system, lung, lymph nodes | Negative | Negative | Yes | No | No | 3.69 | 4.64 | 897.70 | 23493 | 12.85 | No |
| 25 | Female | 1.09 | skin, liver, spleen, hematopoietic system, lung | NA | NA | Yes | Yes | Yes | 2.05 | 1.04 | 358.40 | 20107 | 13.89 | Yes |
| 26 | Female | .73 | Bones, skin, liver, spleen, hematopoietic system, CNS | NA | NA | Yes | Yes | Yes | 1.72 | 1.53 | 516.30 | 12635 | 15.75 | No |
| 27 | Male | .81 | skin, liver, spleen, hematopoietic system, lung | NA | NA | Yes | Yes | Yes | 2.52 | .90 | 43.40 | 13561 | 11.27 | Yes |
| 28 | Male | 1.47 | Bones, skin, liver, spleen, hematopoietic system | Negative | NA | Yes | Yes | Yes | 3.19 | 1.19 | 25.90 | 28487 | NA | No |

NA: not available

Table S2 Longitudinal evaluation of cell-free BRAF-V600E during dabrafenib treatment

| Time during dabrafenib administration | cell-free BRAF-V600E（%） | | | | | | | | | |
| --- | --- | --- | --- | --- | --- | --- | --- | --- | --- | --- |
|  | Pt.1 | Pt.6 | Pt.7 | Pt.3 | Pt.2 | Pt.9 | Pt.8 | Pt.10 | Pt.5 |  |
| Day 0 | 11 | 7.89 | 10 | 12.68 | 1.297 | 22.52 | 0.92 | 1.4 | 15.25 |  |
| Month 1 | 0.45 | 3.08 | 3.76 | 0.18 | 0.18 | 0.2 | 0.76 | 1.14 | 0.01 |  |
| Month 3 | 0.55 | 1.94 | 6.21 | 0.77 | 0.56 | 0.25 | 0.3 | 0.92 | 0.03 |  |
| Month 6 | 0.4 | 2.77 | 5.17 | 0.38 | 0.1 | 0.05 | 0.32 | 1.14 | 0.09 |  |
| Month 9 | 0.42 | 2.29 | 5.07 | NA | NA | 0.14 | 0.11 | 1 | 0.03 |  |

**
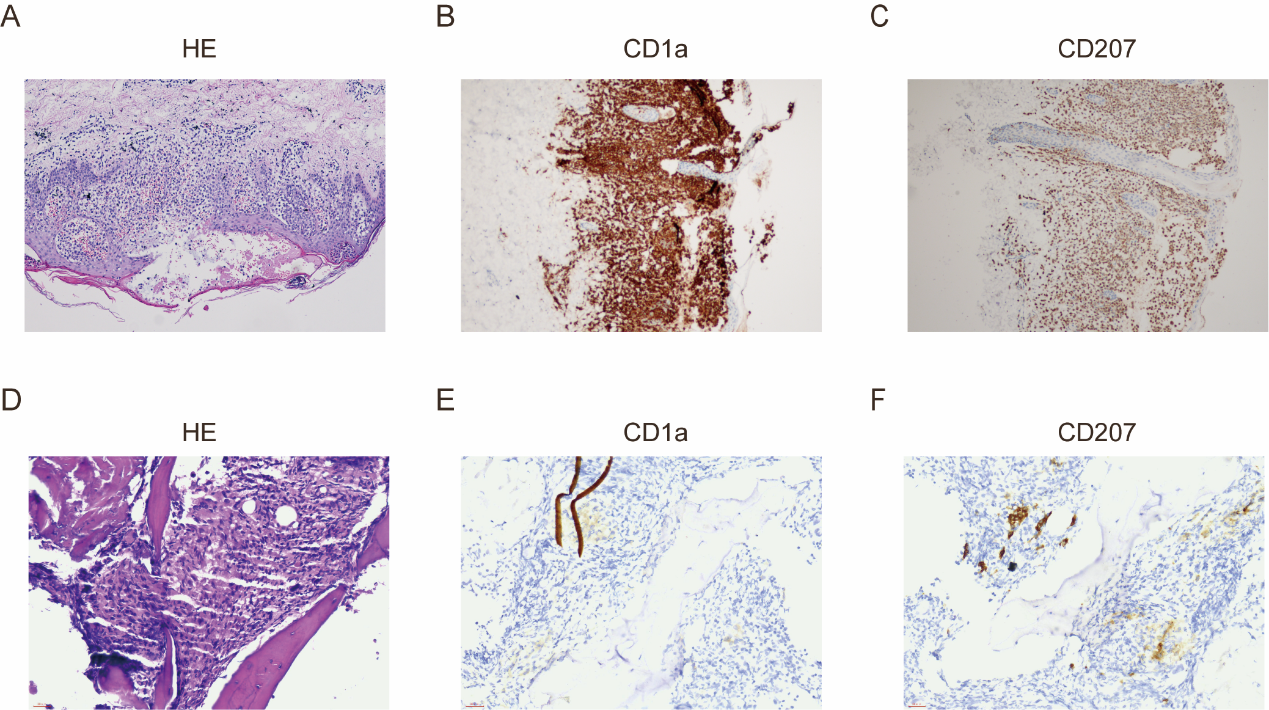
**

Figure S1 Histology of LCH lesions obtained from a skin biopsy (A-C) or a bone marrow biopsy (D-F) in the LCH patients with MAS-HLH. (A) and (D): HE staining; (B) and (E): CD1a-positive immunostaining; (C) and (F): CD207 (langerin)-positive immunostaining.


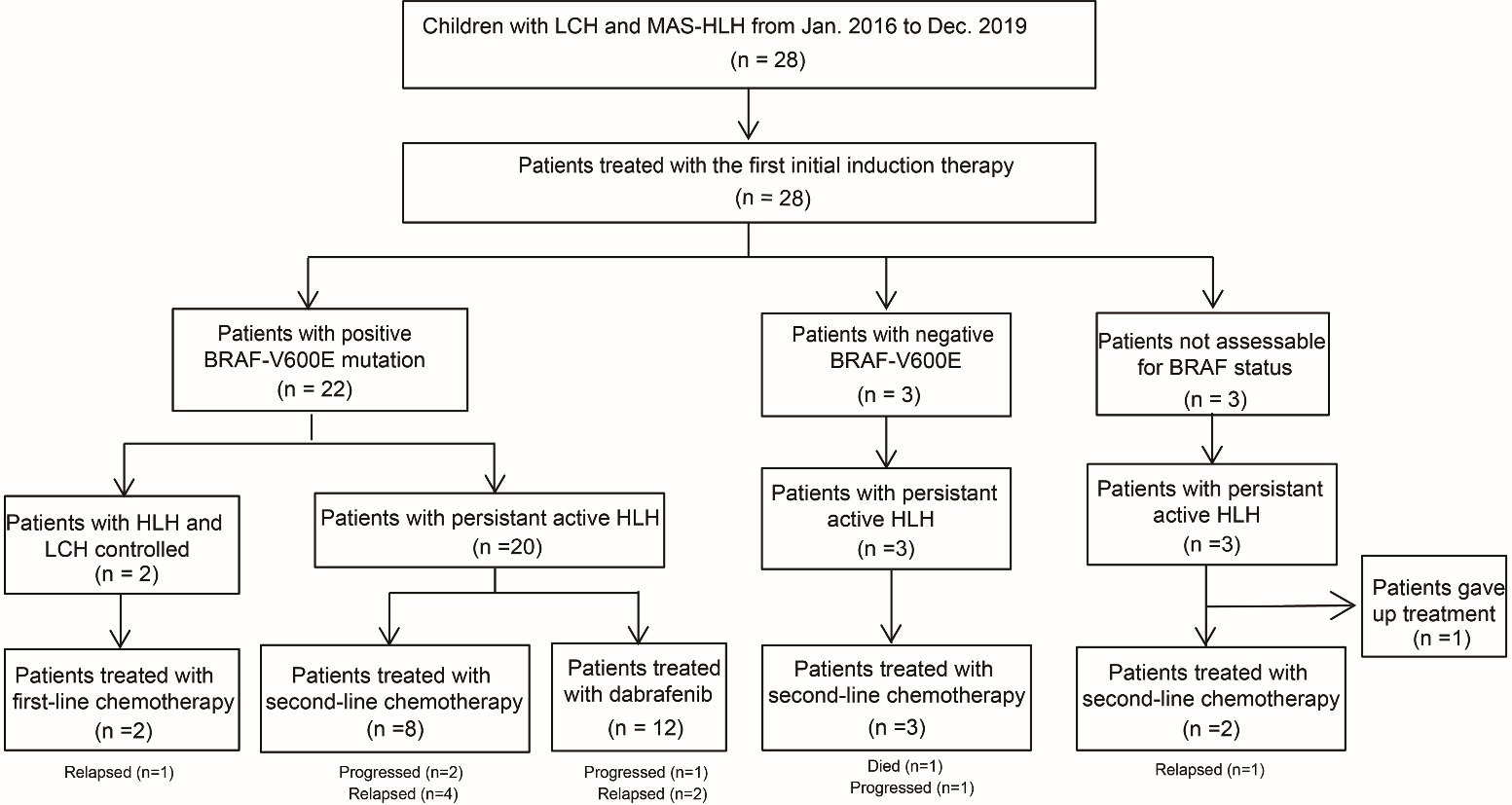


Figure S2 Study cohorts of pediatric LCH and MAS-HLH.


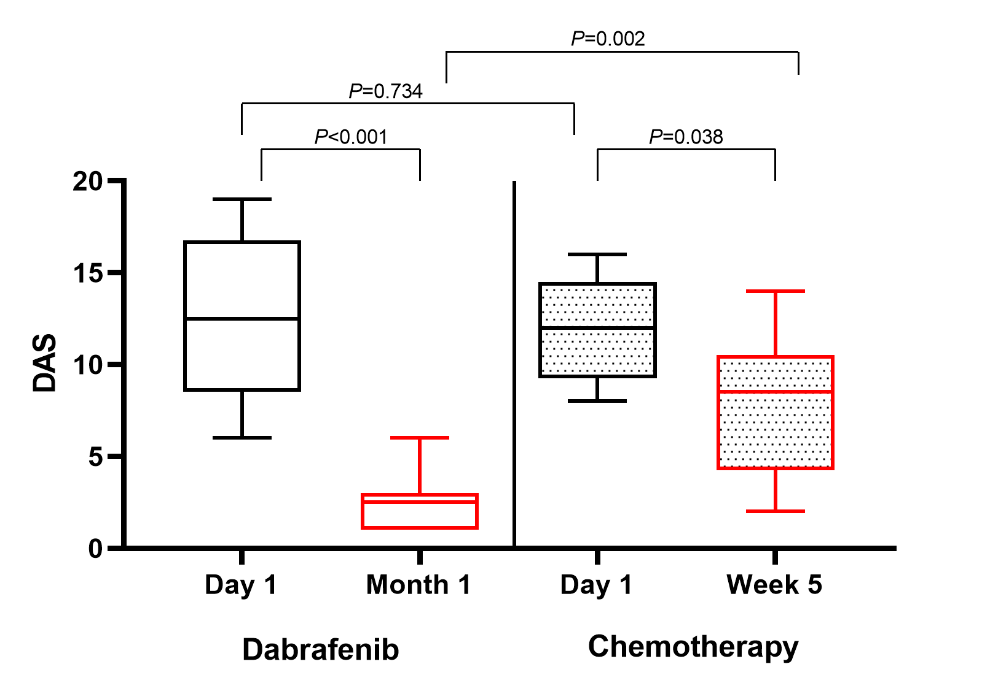


Figure S3 Comparison of DAS after one month of dabrafenib and five weeks (two therapeutic courses) of second-line chemotherapy.
